# FREM1 serves as a novel therapeutic target in breast cancer through basement membrane-based prognostic modeling with integrated bioinformatics and experimental validation

**DOI:** 10.1007/s12672-025-04117-3

**Published:** 2025-12-01

**Authors:** Chao Li, Pingming Gong, Junfeng Hu, Chengyu Luo

**Affiliations:** 1 Department of General Surgery, Beijing Anzhen Hospital, Capital Medical University, Beijing, 100029 China; 2 Department of General Surgery, Beijing Luhe Hospital, Capital Medical University, Beijing, 101149 China

## Abstract

**Background:**

Breast cancer remains the leading cause of cancer-related mortality in women worldwide, with metastatic disease posing significant therapeutic challenges. While immunotherapy has shown promise, tumor immune evasion limits its efficacy. The basement membrane (BM), a specialized extracellular matrix structure, plays a crucial yet understudied role in breast cancer progression and immune modulation. This study aims to investigate the prognostic value and therapeutic potential of BM-related genes in breast cancer.

**Methods:**

We integrated transcriptomic data from TCGA and GEO databases to construct a BM-related gene signature. Unsupervised clustering stratified patients into molecular subtypes, while differential expression analysis identified key BM-associated genes. Functional enrichment analyses (GO, KEGG, GSEA) elucidated biological pathways, and immune microenvironment characterization was performed using ESTIMATE and CIBERSORT. Machine learning approaches pinpointed critical BM-related genes, which were subsequently validated through in vitro experiments.

**Results:**

Breast cancer patients were classified into high- and low-BM groups, with the low-BM cohort exhibiting worse prognosis. Pathway analysis revealed significant enrichment in immune regulation, ECM remodeling, and cytokine signaling. FREM1 emerged as a top protective gene through machine learning. Experimental validation in breast cancer cell lines showed that FREM1 expression was significantly lower in tumor cells compared to normal cells. Upon overexpression of FREM1 in breast cancer cell lines, as confirmed by both qPCR and Western blot, we observed a significant reduction in tumor cell proliferation, migration, and invasion. These findings suggest that FREM1 overexpression impairs the aggressiveness of breast cancer cells, reinforcing its potential as a tumor suppressor.

**Conclusion:**

Our study establishes BM-related genes as novel prognostic biomarkers and therapeutic targets in breast cancer. FREM1 in particular functions as a tumor suppressor by inhibiting cancer cell proliferation, migration, and invasion, highlighting its potential for therapeutic exploitation. These findings provide critical insights into BM-mediated tumor progression and suggest new avenues for targeted breast cancer therapy.

**Supplementary Information:**

The online version contains supplementary material available at 10.1007/s12672-025-04117-3.

## Introduction

Breast cancer (BC) is the most prevalent malignancy among women worldwide, with an estimated 2.3 million new cases diagnosed each year [1], making it the leading cause of cancer incidence globally. Breast cancer is the leading cause of cancer-related deaths among women globally [2], accounting for the fifth highest mortality rate across all cancers [1]. Breast cancer management currently involves a range of treatments, including surgery [3], chemotherapy [4], radiotherapy [5], endocrine therapy [6], and targeted therapies [7]. Despite these options, their effectiveness is often limited, especially in cases of metastatic breast cancer, where long-term survival outcomes remain poor [8, 9]. Recent advancements in immunotherapy have significantly impacted breast cancer treatment [10], offering particularly promising results for triple-negative breast cancer (TNBC) [11] and HER2-positive breast cancer [12]. The emergence of immune checkpoint inhibitors has opened new avenues for strengthening localized anti-tumor immune responses [13]. Nonetheless, while certain patients experience notable clinical benefits, the majority continue to face challenges of disease progression, largely attributed to the development of primary or acquired resistance [14]. Enhancing treatment efficacy requires a deeper understanding of the interaction between breast cancer cells and the immune microenvironment, along with the mechanisms underlying immune evasion. Additionally, the discovery of reliable biomarkers can significantly advance the development of immunotherapy strategies.

The extracellular matrix (ECM) is central to the tumor microenvironment, with the basement membrane (BM) acting as a barrier that prevents cancer cells from spreading [15]. Abnormal regulation of the BM can, therefore, facilitate tumor invasion and metastasis [16]. Several studies have demonstrated that basement membrane-related genes are linked to the prognosis of various cancers, including Lung adenocarcinoma [17] and hepatocellular carcinoma [18]. An emerging study indicate that the progression of breast cancer is driven not only by the tumor cells themselves but also by a significantly altered tumor microenvironment [19]. The extracellular matrix (ECM), particularly the basement membrane (BM), is pivotal in regulating various aspects of tumor biology, including cell migration and invasion [19]. Alterations in the ECM, like collagen IV breakdown, commonly induced by immune cells and cancer-associated fibroblasts, can create a pathway for tumor cells to invade through the basement membrane [20]. These alterations point to a potential yet underexplored connection between the BM and tumor immune infiltration.

In this study, we integrated bioinformatics analyses with experimental validation to investigate the role of FREM1 as a prognostic biomarker and therapeutic target in breast cancer by developing a BM-related gene signature. Our study demonstrated that FREM1 is downregulated in breast cancer tissues compared to normal controls, correlates with poor clinical outcomes and an immunosuppressive tumor microenvironment, and that its overexpression can suppress tumor malignant behaviors.

## Materials and methods

### Data collection and processing

The breast cancer (BRCA) RNA-seq transcriptome data and clinical information were obtained from The Cancer Genome Atlas (TCGA, https://cancergenome.nih.gov/) as the primary dataset. To align with the focus of our study on female breast cancer, we exclusively retained samples from female patients and excluded all male cases. To ensure the robustness of survival analysis, samples lacking survival data were excluded. The data were downloaded in fragments per kilobase million (FPKM) format for consistency in downstream analyses. For external validation, we utilized the GSE131769 dataset from the Gene Expression Omnibus (GEO, http://www.ncbi.nlm.nih.gov/geo), which provides survival information and clinical outcomes. Additionally, two single-cell RNA-seq datasets, GSE148673 and GSE176078, were acquired for investigating the expression patterns of target genes within the breast cancer microenvironment. Based on a thorough review of the literature, we also compiled a list of 222 basement membrane-related genes for further analysis in this study [15].

### Molecular subtyping via consensus clustering

To delineate clinically relevant molecular subtypes based on basement membrane gene expression patterns, we implemented an unsupervised consensus clustering approach [21]. The analysis was conducted using the "ConsensusClusterPlus" R package with the following parameters: 100 resampling iterations to ensure robust cluster stability, a pItem value of 0.8 to maintain sample assignment consistency, and Euclidean distance metric with hierarchical clustering. Systematic evaluation of the consensus matrix and cumulative distribution function curves revealed optimal separation into two distinct molecular subtypes, which we further characterized as ​​basement membrane-high (C1)​​ and ​​basement membrane-low (C2)​​ subgroups based on their relative expression levels of BM components. These subtypes were ​​not predefined clinical categories​​ (e.g., Luminal A/B, HER2 + , or Triple-negative) but were derived exclusively from BM gene expression profiles. The clinical relevance of these BM-defined subtypes was confirmed by survival analysis: Kaplan–Meier curves demonstrated significant differences in overall survival between the C1 (BM-high) and C2 (BM-low) groups (log-rank p < 0.05). This finding underscores the prognostic value of BM gene signature-based stratification in breast cancer.

### Differential expression and functional enrichment analysis

To characterize the molecular distinctions between the identified breast cancer subtypes, we performed differential gene expression analysis using the "limma" package in R, applying thresholds of |logFC|> 1 and adjusted p-value < 0.05. Significantly differentially expressed genes (DEGs) were then functionally annotated using the "clusterProfiler" package. Gene Ontology (GO) [22] analysis categorized DEGs into biological processes, cellular components, and molecular functions, while KEGG (Kyoto Encyclopedia of Genes and Genomes) [23] pathway analysis identified enriched signaling pathways. Additionally, Gene Set Enrichment Analysis (GSEA) [24] was conducted using the "c5.go.symbols.gmt" and "c2.cp.kegg.symbols.gmt" databases from the MSigDB collection to further validate pathway-level associations. This integrated approach provided a systematic framework for interpreting the biological relevance of subtype-specific gene signatures.

### Analysis of tumor microenvironment components

To characterize the tumor microenvironment across molecular subtypes, we employed a comprehensive analytical approach. First, we calculated ESTIMATE (Est​​imation of ​​St​​romal and ​​Imm​​une cells in ​​M​​alignant ​​T​​umor tissues using ​​E​​xpression data) scores to quantify stromal and immune cell infiltration levels as well as tumor purity using standardized parameters [25]. Next, we performed immune cell deconvolution using the CIBERSORTx (C​​ell ​​I​​dentity ​​B​​y ​​E​​stimated ​​R​​egression ​​S​​orting ​​O​​f ​​R​​NA ​​T​​ranscripts) algorithm (v1.06) with the LM22 signature matrix and 1000 permutations to estimate the relative proportions of 22 immune cell subtypes [26], applying a significance threshold of p < 0.05 for reliable detection. Additionally, we systematically evaluated the expression profiles of immunomodulatory markers, including HLA (​​H​​uman ​​L​​eukocyte ​​A​​ntigen) gene family members and key immune checkpoint molecules, using one-way ANOVA with Benjamini–Hochberg correction for multiple testing. This integrated analysis enabled quantitative assessment of the tumor-immune interface characteristics across different molecular subtypes.

### Development and validation of a basement membrane-related prognostic signature

To establish a robust prognostic signature, we first performed univariate Cox regression analysis to identify basement membrane (BM)-related genes significantly associated with overall survival (p < 0.05). These candidate genes were subsequently subjected to Lasso regression with tenfold cross-validation to prevent overfitting and generate a refined risk score model. Patients were dichotomized into high- and low-risk groups using the median risk score as the cutoff value. The model's predictive performance was systematically evaluated through Kaplan–Meier survival curves and risk score distribution visualization. External validation was conducted using an independent GEO cohort to confirm clinical applicability.

To enhance biomarker discovery, we integrated machine learning approaches by combining Lasso regression [27] with support vector machine (SVM) [28] recursive feature elimination. This dual-selection strategy identified consensus prognostic genes exhibiting both statistical significance and biological relevance. The machine learning framework enabled detection of complex genomic patterns that conventional statistical methods might miss, thereby improving the model's predictive accuracy for personalized treatment stratification.

### Comprehensive analysis of key genes in breast cancer expression prognosis and tumor microenvironment

To refine the selection of clinically relevant genes, we systematically analyzed the machine learning-derived candidate genes using TCGA data. We initially conducted Kaplan–Meier survival analysis to investigate potential correlations between gene expression patterns and clinical outcomes in breast cancer patients. We subsequently examined differential gene expression between tumor and adjacent normal tissues to elucidate their possible involvement in breast cancer pathogenesis. This sequential analytical strategy—progressing from clinical relevance to mechanistic exploration—enabled systematic identification of the most promising candidate genes for further investigation. Furthermore, we performed single-cell RNA sequencing analysis to delineate the precise cellular localization and expression patterns of these genes across different cell populations in breast tumors. This comprehensive approach provided critical insights into the potential functional roles of these genes in tumor biology and disease progression.

### Cell line acquisition and culture conditions

We utilized two breast cell lines for our experiments: the highly metastatic MDA-MB-231 cancer cells and non-tumorigenic MCF-10A normal epithelial cells, both obtained from Procell Life Science & Technology Co., Ltd (Wuhan, China). These cell lines were maintained under standard culture conditions (37 °C, 5% CO_2_) using distinct growth media formulations. The MDA-MB-231 line was propagated in high-glucose DMEM supplemented with 10% FBS and antibiotic solution. The MCF-10A normal epithelial cells required a specialized DMEM/F12-based medium containing 5% equine serum along with specific growth factors including EGF (20 ng/mL), hydrocortisone (0.5 μg/mL), insulin (10 μg/mL), and NEAA supplementation.

### Plasmid construction and transfection

Plasmid construction and transfection were performed as previously described [29]. The FREM1 coding sequence was PCR-amplified from MDA-MB-231 cell cDNA using specific primers containing KpnI and XhoI restriction sites (Forward: 5'-GCCGGTACCGCCACCATGGTGACACAAGAATCCATGCTG-3'; Reverse: 5'-GGCCTCGAGTTACTTGTCATCGTCGTCCTTGTAATCGAGTTTTCTGGAACACAC-3'). The amplification was carried out for 30 cycles under standard conditions: 95 °C for 15 s, 68 °C for 15 s, and 72 °C for 1 min per cycle. Following amplification, the PCR product was digested with the corresponding restriction enzymes (Transgen, Beijing, China) at 37°C for 30 min to generate compatible ends for subsequent cloning. The resulting FREM1 fragment was ligated into the ​​pcDNA4 myc-His(A) vector (Invitrogen, Thermo Fisher Scientific)​​, which contains a CMV promoter. The ligation product was transformed into ​​chemically competent E. coli DH5α cells (Takara Bio, Japan)​​ for plasmid amplification. For transfection, ​​1 μg of purified plasmid DNA​​ was introduced into ​​MDA-MB-231 cells​​ using ​​Lipo8000 (Beyotime, Shanghai, China)​​ following the manufacturer’s protocol, with optimization for 6-well plates.

### Quantitative PCR analysis for cell line screening and transduction validation

Quantitative real-time PCR (qPCR) was performed to evaluate the differential expression of the target gene between tumor and normal cell lines and to confirm successful gene introduction post-transfection, as previously described [30]. Total RNA was extracted from cell lines using TRIzol reagent (Servicebio) followed by DNase I treatment to eliminate genomic DNA contamination. cDNA synthesis was carried out with 1 μg total RNA using HiScript II Reverse Transcriptase (Servicebio) and oligo (dT) primers.

For qPCR amplification, reactions were prepared in triplicate with TB Green Premix Ex Taq II (Servicebio) and run on a LightCycler 480 system (Roche) under the following conditions: 95 °C for 30 s, followed by 40 cycles of 95 °C for 5 s and 60 °C for 30 s.

FREM1 primers (Forward: 5'- GACCGAATATGAAGTCTGTGAG-3'; Reverse: 5'- CAGAATTACTTCAAAGACCTC-3') were designed span exon-exon junctions. GAPDH primers (Forward: 5'-CCACTCCTCCACCTTTGAC-3'; Reverse: 5'- ACCCTGTTGCTGTAGCCA-3') served as an endogenous control. Relative expression levels were calculated using the 2^−ΔΔCt^ method. All experiments were conducted with three independent biological replicates, and results are presented as mean ± SD.

### Western blot analysis for FREM1 expression screening and overexpression validation​​

​​Following published procedures for protein validation [29], we implemented Western blot analysis to examine FREM1 expression at the protein level, complementing our transcriptomic data. Total proteins were extracted from breast cancer cell line and normal epithelial cell line, and transduced cells using ice-cold RIPA lysis buffer (Servicebio) supplemented with protease inhibitors. Following centrifugation at 15,000 × g for 30 min at 4 °C, protein concentrations were determined using the BCA Protein Assay Kit (Biosharp).

For FREM1 detection, 10 μL of total protein per sample was separated on 10% SDS-PAGE gels (Servicebio) and transferred to 0.45 μm PVDF membranes (Millipore Sigma). After blocking with 5% non-fat milk in TBST for 2 h at room temperature, membranes were incubated overnight at 4 °C with primary antibodies: anti-FREM1 (1:1000, Proteintech, 13,086–1-AP) and anti-GAPDH (1:5000, Proteintech, 60,004-1-Ig) as loading control. Following three washes with TBST, membranes were incubated with HRP-conjugated secondary antibodies (1:5000, ZSGB-BIO, ZB-2301) for 1 h at room temperature.

Protein signals were detected using the SuperPico ECL Chemiluminescent Substrate (Servicebio) and quantified using ImageJ software (NIH). Successful FREM1 overexpression was confirmed by ≥ 1.5-fold increase in protein expression compared to vector controls (p < 0.05), with all experiments performed in five independent biological replicates.

### EdU and Ki-67 immunofluorescence analysis of FREM1-mediated proliferation inhibition in breast cancer cells

To assess the effect of FREM1 overexpression on breast cancer cell proliferation, we performed EdU incorporation [31] and Ki-67 immunofluorescence [32] assays. For EdU labeling, cells were seeded on coverslips in 48-well plates at a density of 5 × 10^4 cells/well and cultured for 8 h. The cells were then incubated with 10 μM EdU (MCE) for 2 h at 37 °C, fixed with 4% paraformaldehyde for 30 min, and processed using the Click-iT EdU Alexa Fluor 594 Imaging Kit according to the manufacturer's protocol.

For Ki-67 detection, cells grown on coverslips were fixed with 4% paraformaldehyde, permeabilized with 0.3% Triton X-100, and blocked with 5% Donkey Serum. The cells were then incubated overnight at 4 °C with anti-Ki-67 antibody (Abclonal, A20018; 1:400 dilution), followed by incubation with Alexa Fluor 594-conjugated secondary antibody for 2 h at room temperature. Nuclei were counterstained with DAPI (1 μg/mL) for 5 min.

Fluorescence images were acquired using a confocal microscope (Leica TCS SP8), with at least five random fields captured per sample. The proliferation index was calculated as the percentage of EdU-positive or Ki-67-positive cells relative to the total number of DAPI-stained nuclei. Five independent biological replicates were performed for the EdU assay, and four independent biological replicates were performed for the Ki-67 assay.

### Colony formation assay for FREM1-mediated proliferation inhibition in breast cancer cells

To assess how FREM1 overexpression influences breast cancer cell proliferation, we performed a colony formation assay following established protocols [33]. MDA-MB-231 cells were transiently transfected with the FREM1 overexpression plasmid or empty vector control 24 h prior to plating, without using a stable expression vector or antibiotic selection and subsequently seeded into 6-well plates at a density of 1000 cells per well. Cells were cultured under standard conditions (37 °C, 5% CO₂) for 14 days, with the medium replaced every 3 days. When visible colonies appeared, the cells were gently washed with PBS, fixed with 4% paraformaldehyde for 30 min, and stained with 0.5% crystal violet for 30 min at room temperature. Colonies were defined as clusters size ≥ 5 μm and ≤ 10,000 μm, only colonies meeting this threshold were included in the final analysis to exclude small, non-specific cell aggregates and ensure reproducibility. The colony formation efficiency was calculated by normalizing the number of colonies to the number of seeded cells. All assays were repeated five times independently.

### Scratch wound healing assay for FREM1-mediated migration inhibition in breast cancer cells

Consistent with established approaches for measuring cell migration [34], we implemented a scratch wound healing assay to assess FREM1's role in breast cancer cell motility. Briefly, MDA-MB-231 cells were seeded in 6-well plates at a density of 1 × 10^^6^ cells/well and cultured until they reached 90% confluence. A linear scratch was made across the cell monolayer using a sterile 200 μL pipette tip. Cells were washed with PBS to remove detached cells, and fresh medium was added. Images were captured at 0, 24, and 48 h post-scratch using an inverted microscope (Leica) to observe cell migration into the wound area. The migration rate was calculated by measuring the wound closure area, and the percentage of wound closure was determined by comparing the initial and final wound areas. Experiments were repeated five times.

### Transwell invasion assay for FREM1-mediated invasion inhibition in breast cancer cells

To assess the invasive potential of MDA-MB-231 cells after FREM1 overexpression, we performed a Transwell invasion assay as previously described [35], with minor modifications. Briefly, Matrigel-coated Transwell inserts (Corning, USA) were placed in 24-well plates, and 2 × 10^^5^ cells were seeded in the upper chamber with serum-free medium. The lower chamber was filled with complete medium containing 10% FBS to serve as a chemoattractant. After 24 h of incubation at 37°C, the cells that migrated through the Matrigel-coated membrane to the lower surface of the insert were fixed with methanol, stained with crystal violet, and counted in five random fields using an inverted microscope (Leica). ImageJ was used for cell counting. Statistical significance was determined using one-way ANOVA. A value of *p* < 0.05 was considered significant.

### Statistical analysis

All data analyses were conducted with R statistical software (v4.4.0). Continuous variables were analyzed using appropriate tests based on data distribution characteristics: parametric comparisons employed Student's t-test (two groups), One-way ANOVA were used for multiple groups’ comparison and Bonferroni’s post hoc analyses were used to assess the significance between isolated groups. While nonparametric analyses utilized the Wilcoxon rank-sum test (two groups) or Kruskal–Wallis test (multiple groups). Bivariate associations were examined through correlation analysis, with Pearson's coefficient applied to normally distributed variables and Spearman's rank correlation used for non-normal distributions. Survival analysis was evaluated via Kaplan–Meier methodology, with between-group differences tested using the log-rank statistic. All tests were two-sided, and significance was set at p < 0.05 unless otherwise specified.

## Results

### Identification and characterization of basement membrane-based molecular subtypes in breast cancer

**Fig. 1:**
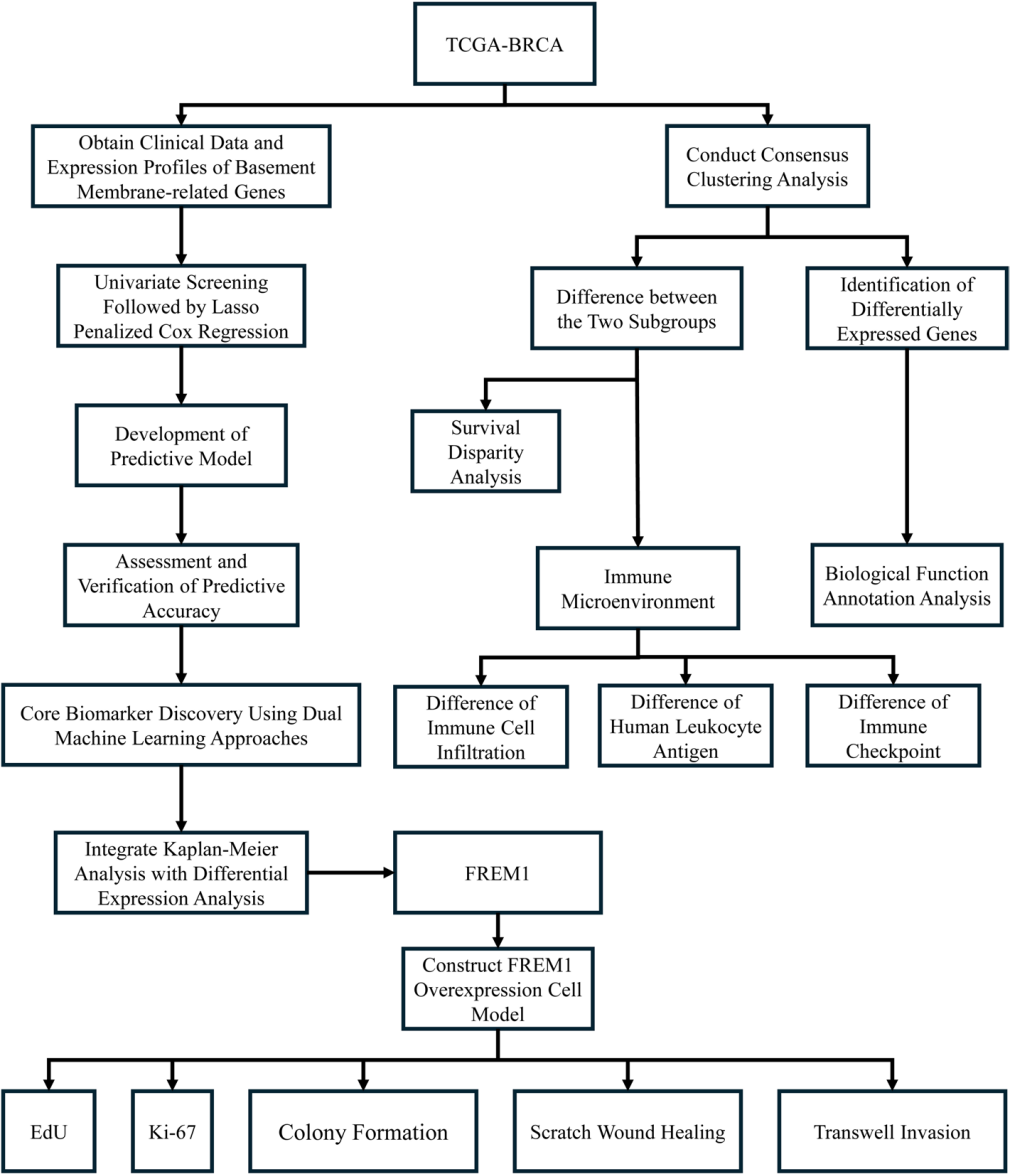
Flowchart of the study design

**Fig. 2:**
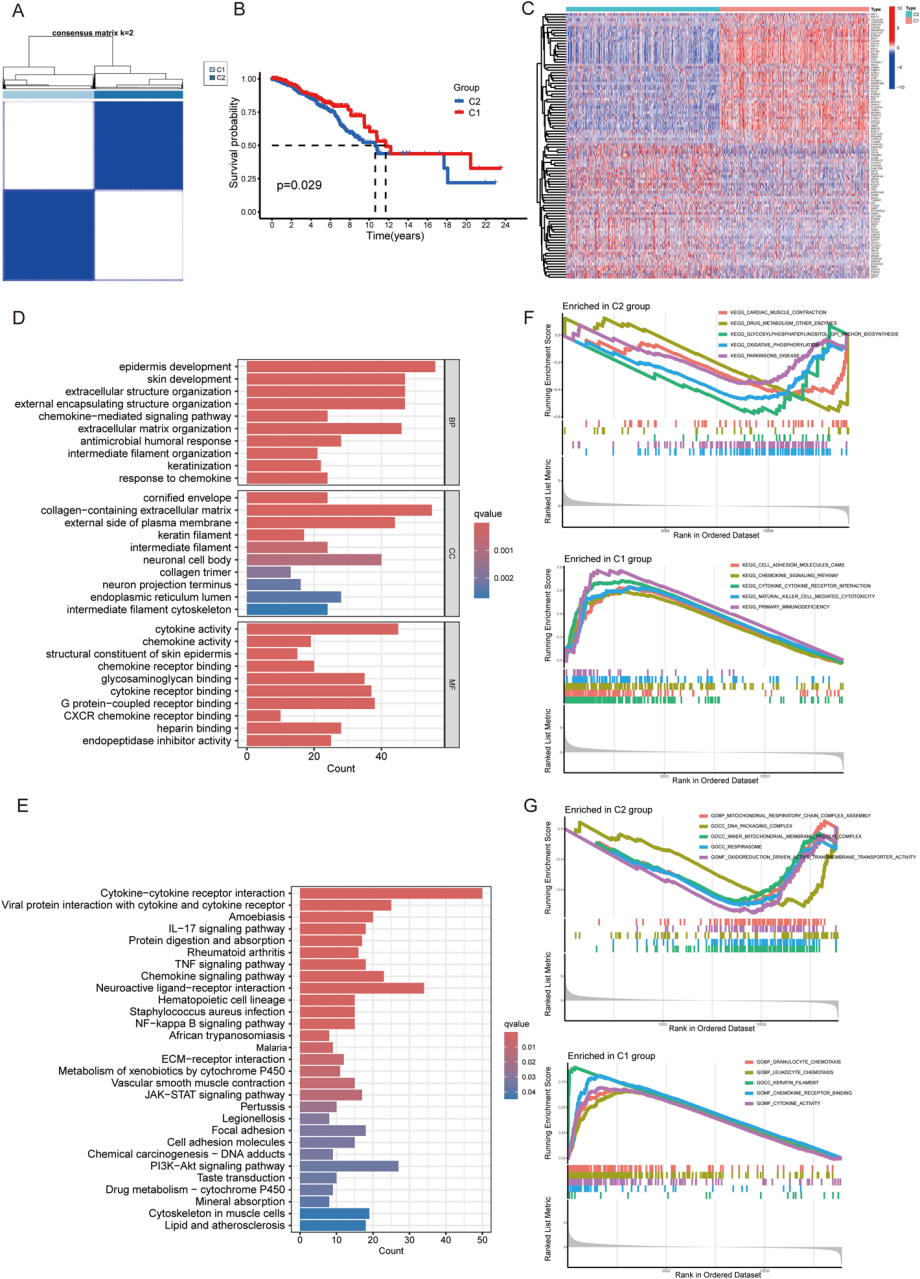
Molecular subtyping and functional characterization of basement membrane-related genes in breast cancer. **A** Unsupervised clustering of TCGA-BRCA samples based on 222 basement membrane-related genes identified two distinct molecular subtypes, C1 (high expression level of BM-related genes) and C2 (low expression level of BM-related genes), demonstrating significant transcriptional heterogeneity. **B** Kaplan–Meier survival analysis revealed markedly different clinical outcomes between subtypes (log-rank p < 0.05, n = 1,097), establishing the prognostic significance of basement membrane gene expression patterns. **C** Hierarchical clustering heatmap visualized the differential expression profiles of top 50 basement membrane-related genes across subtypes. **D**-**G** Functional enrichment analyses (GO, KEGG, and GSEA) of differentially expressed genes revealed significant associations with extracellular matrix remodeling and immune regulation processes. Enrichment significance was determined by false discovery rate (FDR) < 0.05

Figure 1 outlines our analytical workflow (Fig. 1). Consensus clustering of TCGA-BRCA samples using 222 basement membrane-related genes robustly segregated patients into two subtypes: a basement membrane-high group (C1) showing elevated expression of core components, and a basement membrane-low group (C2) with significantly reduced expression (Fig. 2A). Kaplan–Meier analysis demonstrated significantly poorer overall survival in the C2 (basement membrane-low) versus C1 (basement membrane-high) group (p = 0.029, Fig. 2B). The hierarchical clustering heatmap (Fig. 2C) displays the expression profiles of the top 50 most significantly differentially expressed genes (|log2FC|> 1, FDR < 0.05) between C1 and C2 subtypes, revealing distinct molecular signatures characteristic of each subgroup.

### Functional annotation of BM-associated DEGs uncovers their involvement in breast cancer microenvironment

To investigate the biological significance of differentially expressed genes (DEGs) across basement membrane-associated subgroups, we performed integrated functional enrichment analyses including Gene Ontology (GO), Kyoto Encyclopedia of Genes and Genomes (KEGG), and Gene Set Enrichment Analysis (GSEA). The GO analysis revealed significant enrichment in Biological Processes, with key pathways including epidermis and skin development, extracellular matrix and structure organization, chemokine-mediated signaling, antimicrobial humoral response, keratinization, and intermediate filament organization (Fig. 2D). In the Cellular Component category, notable enrichments were observed in the cornified envelope, collagen-containing extracellular matrix, keratin and intermediate filaments, neuronal cell body, endoplasmic reticulum lumen, and neuron projection terminus (Fig. 2D). Molecular Function analysis identified associations with cytokine and chemokine activity, structural constituents of skin epidermis, chemokine receptor binding, glycosaminoglycan binding, G protein-coupled receptor binding, and endopeptidase inhibitor activity (Fig. 2D).

KEGG pathway analysis revealed the top 10 significant pathways, including Cytokine-cytokine receptor interaction, Viral protein interaction with cytokine and cytokine receptor, Amoebiasis, IL-17 signaling pathway, Protein digestion and absorption, Rheumatoid arthritis, TNF signaling pathway, Chemokine signaling pathway, Neuroactive ligand-receptor interaction, and Hematopoietic cell lineage, as shown in Fig. 2E.

To identify pathway-level differences between BM expression subgroups, we conducted gene set enrichment analysis (GSEA) utilizing the Molecular Signatures Database (MSigDB) collections, including the GO gene sets (c5.go.symbols.gmt) and canonical KEGG pathways (c2.cp.kegg.symbols.gmt). In the C2 group, significant enrichment was observed in several immune-related KEGG pathways, including Cardiac Muscle Contraction, Drug Metabolism Other Enzymes, Glycosylphosphatidylinositol GPI Anchor Biosynthesis, Oxidative Phosphorylation, and Parkinson’s Disease. Additionally, the C2 group showed enrichment in GO terms such as Mitochondrial Respiratory Chain Complex Assembly, DNA Packaging Complex, Inner Mitochondrial Membrane Protein Complex, Respirasome, and Oxido-reduction Driven Active Transmembrane Transporter Activity (Fig. 2F, G). In contrast, the C1 group was significantly enriched in KEGG pathways such as Cell Adhesion Molecules, Chemokine Signaling Pathway, Cytokine-Cytokine Receptor Interaction, Natural Killer Cell-Mediated Cytotoxicity, and Primary Immunodeficiency. The C1 group also showed enrichment in GO terms such as Granulocyte Chemotaxis, Leukocyte Chemotaxis, Keratin Filament, Chemokine Receptor Binding, and Cytokine Activity. These analyses suggest that basement membrane-related genes may play a role in modulating breast cancer microenvironment and metabolism. These analyses suggest that basement membrane-related genes may influence the breast cancer microenvironment and metabolism (Fig. 2F, G).

### Comprehensive immune profiling revealed significant differences in the tumor microenvironment between BM-defined subgroups

**Fig. 3:**
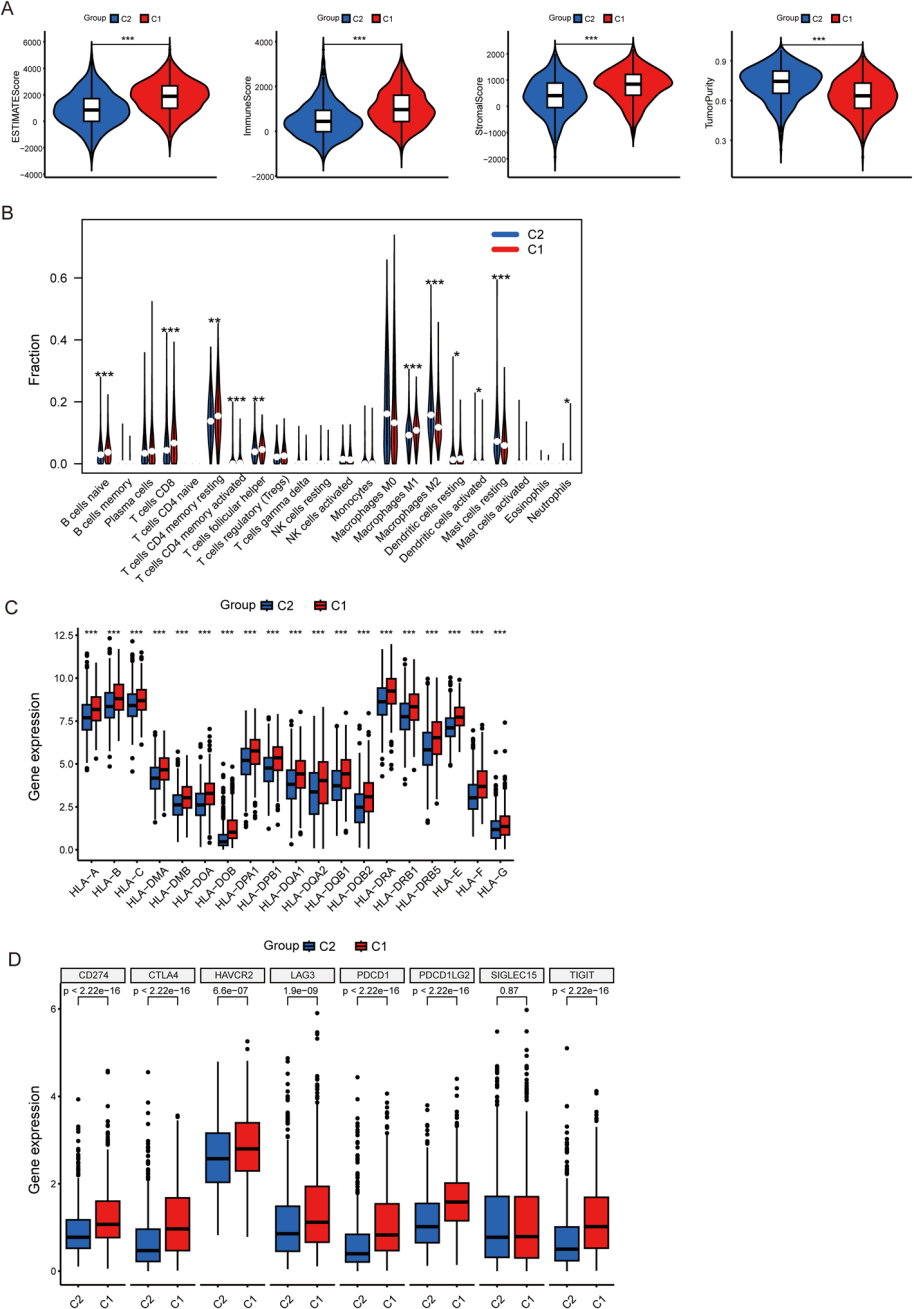
Tumor-immune microenvironment disparities between basement membrane-defined subgroups in breast cancer. **A** Comparative assessment of tumor microenvironment components using ESTIMATE algorithm-derived scores. **B** Differential infiltration patterns of 22 immune cell subtypes identified by CIBERSORT analysis. **C** Distinct expression profiles of HLA (Human Leukocyte Antigen) genes, indicating altered immunogenicity between clusters. **D** Cluster-specific immune checkpoint expression patterns associated with potential evasion mechanisms

The C1 group demonstrated elevated ESTIMATE, immune, and stromal scores alongside reduced tumor purity compared to the C2 group (Fig. 3A). Further analysis using CIBERSORT revealed substantial differences in the infiltration levels of various immune cells between the two basement membrane groups. These included naïve B cells, CD8 + T cells, memory resting CD4 + T cells, memory activated CD4 + T cells, follicular helper T cells, M1 macrophages, M2 macrophages, resting and activated dendritic cells, resting mast cells, and neutrophils (Fig. 3B). These results highlight the crucial role of the basement membrane in influencing the breast cancer immune microenvironment. Furthermore, we observed significant differential expression of HLA genes and immune checkpoint molecules between the groups (Fig. 3C, D), highlighting the crucial role of basement membrane components in shaping the breast cancer immune landscape. These findings collectively demonstrate that BM-related gene expression patterns significantly influence both the cellular composition and immunoregulatory characteristics of the tumor microenvironment.

### Construction and multi-dimensional validation of a prognostic BMS model in breast cancer

**Fig. 4:**
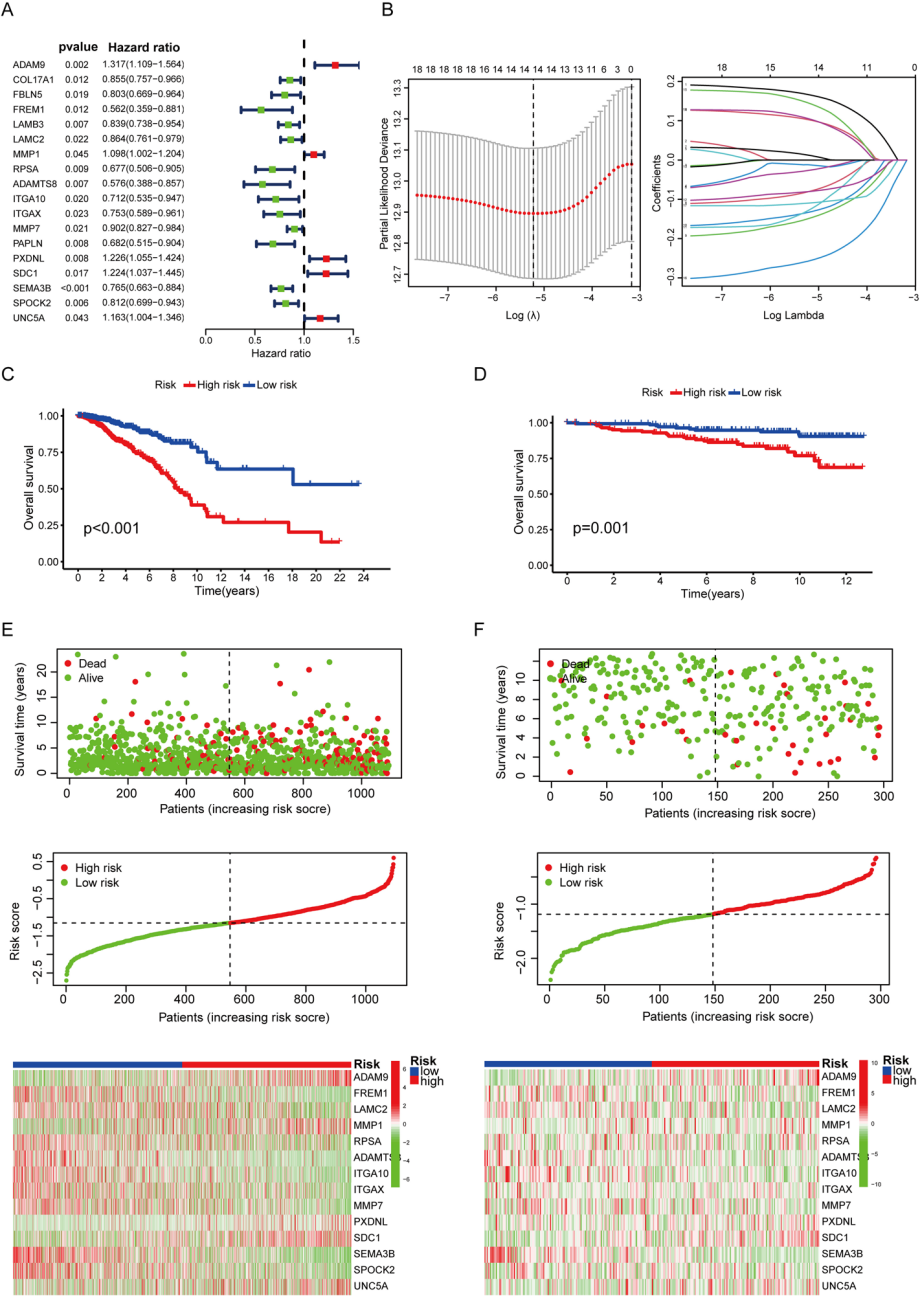
Construction and validation of the basement membrane-related prognostic signature in breast cancer. **A** Univariate Cox regression of potential biomarkers. Hazard ratios (HR), 95% confidence intervals (CI), and p-values shown. **B** LASSO regression-derived 14-gene signature. **C**-**D** Survival curves comparing high- and low-risk groups in TCGA (left) (p < 0.001) and GEO (right) (p = 0.001) datasets. **E** Association between risk stratification and clinical outcomes in the TCGA cohort, along with the expression heatmap of the 14-gene signature across high- and low-risk groups. **F** External independent validation of the prognostic model in the GEO cohort

Through univariate Cox regression analysis, we initially identified 18 basement membrane-related genes significantly associated with patient prognosis (Fig. 4A). Subsequent LASSO regression analysis refined this set to 14 key prognostic genes, which were used to construct a predictive model named the Basement Membrane Signature (BMS) (Fig. 4B). Each breast cancer sample was assigned a BMS risk score, with patients dichotomized into high- and low-risk groups based on the median score. Survival analysis confirmed significantly worse outcomes in the high-risk group, with robust reproducibility across both the TCGA (Fig. 4C) training cohort (p < 0.001) and independent GEO (Fig. 4D) validation dataset (p = 0.001), demonstrating the prognostic validity of this signature. Risk score distributions exhibited consistent patterns between TCGA (Fig. 4E) and GEO (Fig. 4F) cohorts, demonstrating the reproducibility of our risk stratification model.

**Fig. 5:**
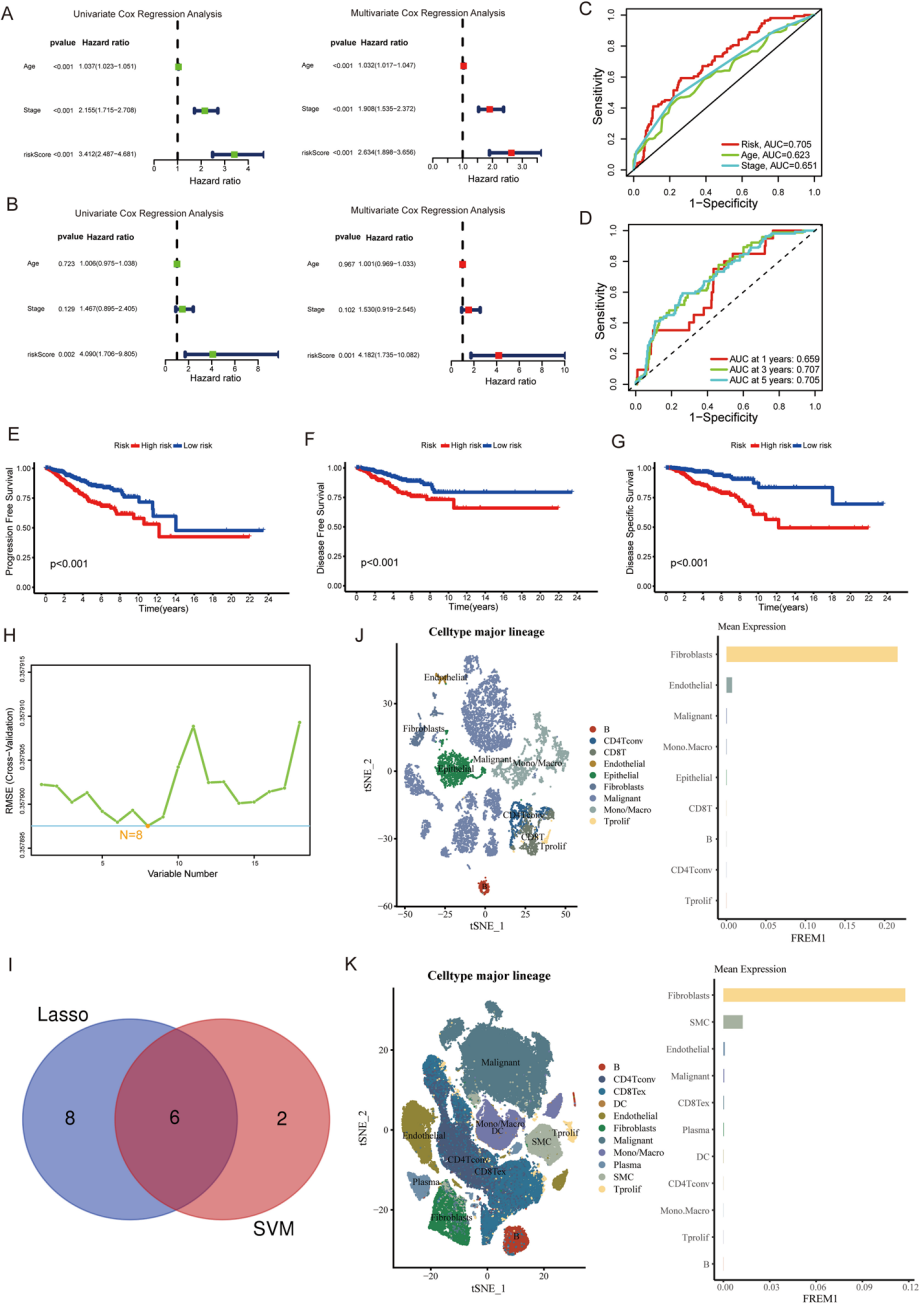
Comprehensive analysis of prognostic factors and tumor microenvironment in breast cancer. **A** Univariate and multivariate Cox regression analyses of TCGA-derived clinical features (Age, Stage, and Riskscore) identifying independent prognostic factors in AML. **B** Validation of prognostic factors through univariate and multivariate Cox regression using GEO database. **C**, **D** ROC curve analyses demonstrated the predictive performance of Age, Stage, and Riskscore, followed by time-dependent evaluation (1-/3-/5-year) using Riskscore alone. **E**–**G** Survival outcomes were compared between high- and low-risk groups, including progression-free survival (PFS) (p < 0.001), disease-free survival (DFS) (p < 0.001), and disease-specific survival (DSS) (p < 0.001). (H) Support vector machine (SVM) algorithm for feature selection. **I** Venn diagram showing overlapping genes identified by both LASSO regression and SVM. **J**, **K** Single-cell RNA-seq analysis of FREM1 expression distribution in breast cancer microenvironment from GSE148673 and GSE176078 datasets

Comprehensive Cox regression analyses revealed distinct prognostic patterns across datasets. In the TCGA cohort, univariate and multivariate analyses consistently identified age, TNM stage, and BMS risk score as significant independent prognostic factors (all p < 0.001, Fig. 5A). Significant differences in age, stage, and prognostic status between the high- and low-risk groups were observed, as shown in Supplementary Table 1. However, validation in the GEO dataset demonstrated that only BMS risk score maintained its prognostic significance (p = 0.001, Fig. 5B), underscoring its robust predictive value across diverse patient populations.

Discriminative ability was assessed through receiver operating characteristic (ROC) analysis. Comparative evaluation of age, TNM stage, and the BMS risk score yielded AUC values of 0.623, 0.651, and 0.705, respectively (Fig. 5C). Time-dependent ROC analysis further demonstrated the stable predictive capacity of the BMS risk score across clinical endpoints, with AUCs of 0.659 (1 year), 0.707 (3-year), and 0.705 (5-year) survival (Fig. 5D).

Analysis of TCGA breast cancer samples demonstrated that patients in the high-risk group had significantly poorer outcomes in progression-free survival (PFS, p < 0.001) (Fig. 5E), disease-free survival (DFS, p < 0.001) (Fig. 5F), and disease-specific survival (DSS, p < 0.001) compared to the low-risk group (Fig. 5G). To further validate these findings, we constructed prognostic nomograms and calibration curves for the predictive models incorporating age, TNM stage and the Basement Membrane Signature (BMS) risk score. These nomograms and calibration curves were developed to assess the predictive accuracy for overall survival (OS) (Supplementary Fig. 1A), progression free survival (PFS) (Supplementary Fig. 1B), disease free survival (DFS) (Supplementary Fig. 1C) and disease specific survival (DSS) (Supplementary Fig. 1D) outcomes. The close alignment between predicted and observed outcomes in both the nomograms and validation curves strongly supported the reliability of these prognostic models.

### Identification and characterization of FREM1 as a prognostic biomarker in cancer

To refine our gene signature, we employed a machine learning approach using support vector machines (SVM), which identified a set of candidate genes (Fig. 5H). Intersection of these SVM-derived candidates with our earlier LASSO-selected genes revealed six core prognostic genes: SEMA3B, ITGAX, FREM1, ADAM9, ADAMTS8, and UNC5A (Fig. 5I). Subsequent validation in the TCGA cohort demonstrated while all four genes (SEMA3B, ITGAX, FREM1, and ADAMTS8) showed significant associations with overall survival (OS) in Kaplan–Meier analysis (log-rank p < 0.05), FREM1 exhibited the most pronounced survival benefit (lowest p-value) (Supplementary Fig. 2A). Differential expression analysis between tumor and adjacent normal tissues revealed that FREM1 showed the most significant downregulation among all candidate genes (p < 0.05), with a consistent protective pattern (Supplementary Fig. 2B). Significant differences in age, stage, and prognostic status were observed between the high and low FREM1 expression groups, as shown in Supplementary Table 2. Given its strongest prognostic performance, most remarkable tumor-specific expression pattern, and putative tumor-suppressive role, we prioritized FREM1 as our primary target for mechanistic investigation.

Finally, using single-cell RNA sequencing data from two independent datasets-GSE148673 (Fig. 5J) and GSE176078 (Fig. 5K)-we performed a comprehensive analysis of FREM1 expression across diverse cell populations within the tumor microenvironment. The results demonstrated that FREM1 was predominantly expressed in cancer-associated fibroblasts (CAFs), with minimal expression in other cell types. This fibroblast-specific expression pattern suggests that FREM1 may play a crucial role in tumor-stroma interactions and extracellular matrix remodeling. Furthermore, we observed that higher levels of FREM1 expression were positively correlated with increased infiltration of CD8 + T cells, a key population of cytotoxic lymphocytes involved in antitumor immunity (Supplementary Fig. 3). In contrast, FREM1 expression appeared to be negatively correlated with immunosuppressive cell populations such as regulatory T cells (Tregs), which are known to dampen anti-tumor immune responses (Supplementary Fig. 3). Together, these findings highlight FREM1's unique role in shaping the tumor microenvironment-not only through its involvement in stromal and matrix-related processes but also potentially through its influence on immune cell composition. This dual function supports the notion that FREM1 may serve as both a stromal biomarker and a potential therapeutic target in breast cancer progression, with implications for both tumor structure and immune modulation.

**Fig. 6:**
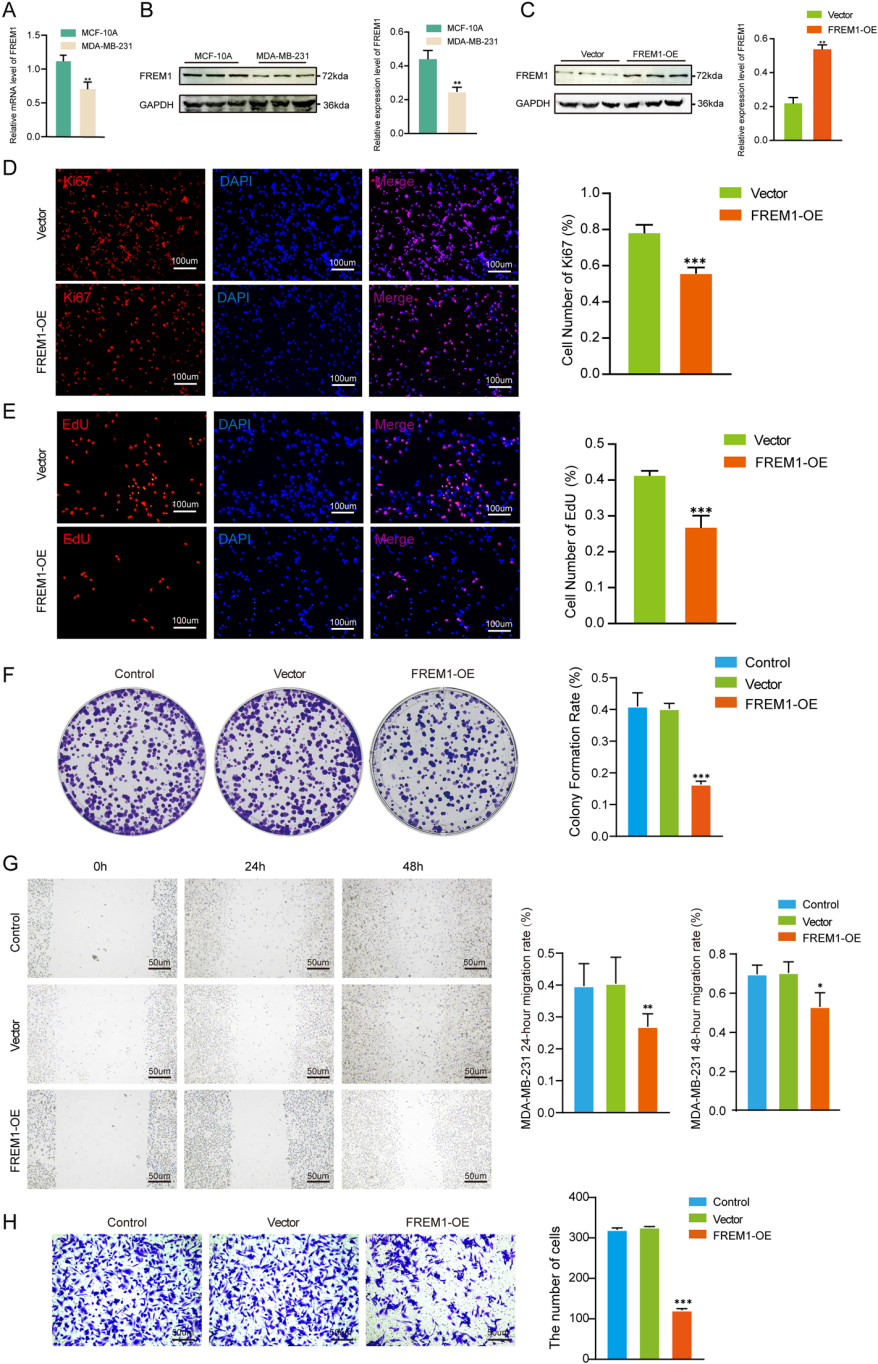
Experimental validation of FREM1 overexpression and functional characterization in breast cancer cells. **A** The mRNA level was higher in MCF-10A than in MDA-MB-231 cell lines (n = 3). **B** The protein level was higher in MCF-10A than in MDA-MB-231 cell lines (n = 5). **C** The protein level was higher in FREM1-overexpressed group than in control group (n = 5). **D** The density of Ki67-positive staining in the FREM1 overexpression group was lower than that in the control group (n = 4). **E** The density of EdU-positive staining in the FREM1 overexpression group was lower than that in the control group (n = 5). **F** Representative images of colony formation showed that the number of cell clones in the FREM1 overexpression group was lower than that in untransfected control and vector groups (n = 5). **G** Scratch wound healing assay showed that the percentage of wound closure in the FREM1 overexpression group was slower than that in untransfected control and vector groups (n = 5). **H** Transwell invasion assay showed that the number of invasive cells in the FREM1 overexpression group was lower than that in untransfected control and vector groups (n = 5)

To functionally characterize FREM1, we performed in vitro studies using triple-negative MDA-MB-231 breast cancer cells. Both qPCR (Fig. 6A) and Western blot (Fig. 6B) analyses confirmed significantly lower FREM1 expression in MDA-MB-231 cells compared with normal mammary epithelial cells (MCF-10A), with > 1.41-fold reduction at the mRNA level (p < 0.01) and > 1.77-fold decrease at the protein level (p < 0.01). Following transfection with a FREM1 overexpression plasmid, MDA-MB-231 cells showed 2.42-fold increased protein expression (p < 0.001) (Fig. 6C) compared with vector controls.

Functional assays consistently demonstrated that FREM1 overexpression significantly suppressed the malignant behavior of breast cancer cells. Ki-67 immunofluorescence (Fig. 6D) revealed 32.6% fewer proliferating cells in the FREM1 overexpression group compared with the vector group (p < 0.001), while EdU assays (Fig. 6E) showed a 34.8% reduction in S-phase cells (p < 0.001). Consistently, colony formation assays (Fig. 6F) demonstrated markedly fewer and smaller colonies in the FREM1 overexpression group, with a 41.2% decrease in colony formation efficiency relative to vector controls (p < 0.001). In scratch wound healing assays (Fig. 6G), both untransfected and vector control cells exhibited rapid migration, whereas the FREM1 overexpression group showed delayed wound closure, with migration reduced by 33.4% at 24 h and 49.2% at 48 h (p < 0.001). Similarly, Transwell invasion assays (Fig. 6H) revealed strong invasive potential in untransfected and vector control cells, while the FREM1 overexpression group exhibited a 62.9% reduction in invasive cell counts compared with vector controls (p < 0.001).

## Discussion

Our study establishes a novel prognostic signature based on basement membrane (BM)-related genes, which effectively stratifies breast cancer patients into distinct risk groups with significant differences in clinical outcomes. This BM-related gene signature (BMS) not only demonstrates robust predictive accuracy for survival but also reveals the critical role of BM components in shaping tumor progression and the immune microenvironment. By integrating multi-omics data and functional validation, we highlight the potential of BMS as a valuable tool for prognosis assessment and personalized treatment strategies in breast cancer.

Breast cancer represents a highly heterogeneous disease with diverse molecular subtypes and clinical outcomes, underscoring the need for precise prognostic biomarkers [36]. Emerging evidence has highlighted the basement membrane (BM) as a dynamic structural and functional component that actively participates in tumor progression and metastasis [37, 38]. Unlike traditional views of the BM as a passive barrier, recent studies demonstrate its crucial role in regulating cancer cell invasion, immune cell infiltration, and therapeutic resistance [19, 39]. The BM's unique composition of collagen IV, laminins, and other glycoproteins forms a specialized extracellular matrix niche that influences tumor-stroma crosstalk and metastatic dissemination [40, 41]. Our identification of BM-related gene signatures builds upon these findings by providing a systematic framework to quantify BM remodeling patterns and their clinical implications. This approach offers new opportunities to understand breast cancer heterogeneity beyond conventional molecular subtyping, potentially guiding more personalized treatment strategies.

Our study systematically evaluated the prognostic value of basement membrane (BM)-related genes in breast cancer through a multi-stage analytical approach. Initially, consensus clustering analysis of BM-related gene expression patterns revealed two distinct molecular subtypes with significant clinical differences. The observed disparity in clinical outcomes between risk groups may be mechanistically linked to distinct immunological characteristics within the tumor microenvironment. These findings suggested that BM composition actively shapes both tumor behavior and immune responses [42, 43].

To refine these observations into a clinically applicable tool, we employed machine learning techniques including LASSO regression and Cox proportional hazards modeling. This process identified a robust 14-gene signature (BMS) that effectively stratified patients by risk across multiple independent cohorts. The signature showed significant prognostic discrimination, with high-risk patients exhibiting substantially worse clinical outcomes than low-risk patients. Importantly, the signature maintained its predictive power even after adjusting for conventional clinical parameters such as age and tumor stage in multivariate analyses. As a multi-gene signature, BMS offers a more comprehensive view of tumor biology. It not only provides robust prognostic stratification but also captures complex biological processes—such as interactions within the immune microenvironment and extracellular matrix remodeling—that are not fully represented by existing single-gene or limited-gene models. These features suggest that BMS has the potential to complement existing genomic signatures (e.g., Oncotype DX, MammaPrint, and PAM50) by providing additional prognostic and biological insights, thereby enhancing the overall understanding and prediction of breast cancer outcomes. The clinical utility of this signature was further enhanced through the development of comprehensive nomograms that integrate BM-related gene expression with standard prognostic factors. These tools showed excellent predictive accuracy for both short-term and long-term outcomes, providing clinicians with a quantitative framework for risk assessment [44, 45]. Mechanistically, the signature captures key biological processes in cancer progression, including extracellular matrix remodeling and Cytokine-cytokine receptor interaction. The strong association between signature scores and specific immune cell populations suggests potential utility in predicting response to immunotherapy.

These findings significantly advance our understanding of BM biology in breast cancer, transforming it from a passive structural component to an active regulator of tumor progression and microenvironment modulation. The signature provides both prognostic value and biological insights, offering opportunities for more personalized treatment approaches. Future studies should explore its potential in guiding therapeutic decisions, particularly for aggressive subtypes where current prognostic tools remain limited. The integration of BM-related gene expression patterns into clinical decision-making represents a promising avenue for improving breast cancer management and outcomes.

To identify clinically relevant targets from our basement membrane-related gene signature (BMS), we employed a machine learning pipeline integrating ​​LASSO regression and support vector machine (SVM) algorithms​​. This approach identified six high-confidence candidate genes, among which ​​FREM1​​ emerged as the most consistently ​​downregulated gene in breast tumor tissues compared to matched normal samples​​. This finding pointed to a potential tumor-suppressive role for FREM1 and underscored its relevance in breast cancer biology. To further investigate its functional significance, we established a ​​FREM1-overexpressing breast cancer cell model​​ and confirmed successful upregulation of both mRNA and protein levels. Functional assays subsequently demonstrated that ​​FREM1 overexpression led to significant suppression of multiple malignant phenotypes​​, including reduced cell proliferation, impaired colony-forming ability, attenuated cell migration, and inhibited invasive capacity. These observations support the notion that FREM1 functions as a key regulator of tumor cell-autonomous behaviors and may act as a potent tumor suppressor in breast cancer.

​​More importantly, our integrative analysis revealed a potential link between FREM1 expression and the composition of the tumor immune microenvironment.​​ Higher levels of FREM1 expression were found to be associated with ​​increased infiltration of cytotoxic CD8 + T cells​​, which play a central role in anti-tumor immune responses. Conversely, FREM1 expression showed an inverse relationship with ​​immunosuppressive cell populations, particularly regulatory T cells (Tregs)​​, which are known to inhibit effective immune surveillance and promote tumor immune evasion. These associations suggest that FREM1 may influence not only the intrinsic behavior of tumor cells but also the surrounding immune landscape, thereby contributing to the shaping of a more immunologically active tumor microenvironment. Taken together, these findings position FREM1 as a multifunctional player in breast cancer biology.​​ Beyond its role in extracellular matrix (ECM) remodeling and stromal interactions, FREM1 appears to be involved in modulating immune cell infiltration and the balance between pro- and anti-tumor immune responses. This dual functionality highlights its potential as both a ​​biomarker with prognostic significance​​ and a ​​therapeutic target with immunomodulatory implications​​.

These experimental findings are supported by accumulating clinical evidence establishing FREM1 as a tumor suppressor in breast cancer. Multiple independent cohorts have confirmed the significant downregulation of FREM1 in malignant versus normal breast epithelium. Importantly, reduced FREM1 expression correlates with aggressive clinicopathological features including advanced TNM stage and poorer survival outcomes, while also associating with an immunosuppressive tumor microenvironment marked by diminished cytotoxic lymphocyte infiltration and increased immunosuppressive cell populations [46, 47].

Notably, our findings align conceptually with a pivotal prior study [47], which similarly reported decreased FREM1 expression in breast cancer tissues relative to normal controls and identified a correlation between low FREM1 levels and unfavorable prognosis, as well as enriched immune cell infiltration. However, our work extends beyond this foundational research by integrating machine learning algorithms to construct a prognostic gene signature, and by conducting comprehensive in vitro functional assays (including proliferation, migration, and invasion) to mechanistically validate FREM1's role in modulating tumor aggressiveness. While the prior study primarily focused on correlative analyses through bioinformatics and immunohistochemistry to explore associations with clinicopathological traits and immune infiltration, our study provides novel experimental evidence demonstrating that FREM1 overexpression suppresses malignant phenotypes, thereby positioning FREM1 as a key regulator of breast cancer progression with both prognostic value and therapeutic potential for overcoming tumor immunosuppression. Collectively, these data underscore FREM1's multifaceted role in breast cancer biology and highlight the advancements contributed by our integrated experimental approach. Collectively, these data position FREM1 as a key regulator of breast cancer progression, with both prognostic implications and potential therapeutic relevance for overcoming tumor immunosuppression.

Our study establishes the Basement Membrane Signature (BMS) as a novel prognostic tool in breast cancer, demonstrating its ability to stratify patients into distinct risk groups with significant differences in clinical outcomes. By integrating multi-omics data and machine learning approaches, we developed a robust 14-gene signature that effectively predicts survival and reflects key biological processes, including extracellular matrix remodeling and immune microenvironment modulation. However, several limitations should be acknowledged. First, our findings are based on retrospective analyses of publicly available datasets, which may be subject to selection biases and technical variability. Although we validated the BMS in both TCGA and GEO cohorts, further confirmation in prospective, multi-center studies is needed to ensure its clinical applicability. Additionally, our study was conducted exclusively in female breast cancer patients, as the datasets utilized (TCGA-BRCA and selected GEO cohorts) predominantly comprised female individuals. The potential relevance of the BMS and FREM1 in male breast cancer (MBC) worth further investigation. Moreover, while we identified FREM1 as a potential tumor suppressor through in vitro experiments, the functional roles of other signature genes in breast cancer progression remain to be fully elucidated. Additional mechanistic studies, including in vivo models and patient-derived xenografts, are required to validate their biological significance and therapeutic potential. Furthermore, breast cancer is a highly heterogeneous disease, and molecular subtypes such as TNBC, HER2-enriched, Luminal A, and Luminal B are known to exhibit distinct biological behaviors, treatment responses, and prognoses. Therefore, it is possible that the ​​predictive value or biological impact of the BMS may vary across subtypes​​, and some subgroups may derive greater benefit from the signature than others. ​​Future studies should validate the BMS in subtype-specific cohorts to determine its utility, generalizability, and potential subtype-specific performance.​​

Despite these limitations, our work provides a foundation for incorporating basement membrane biology into breast cancer prognostication and therapy. The BMS model offers a clinically actionable tool that complements existing prognostic markers, while the identified genes present new opportunities for therapeutic development. Continued validation and functional studies will be essential to translate these findings into improved patient outcomes.

## Conclusion

Our study establishes the Basement Membrane Signature (BMS) as a robust prognostic tool in breast cancer, effectively stratifying patients into distinct risk groups with significant survival differences. By integrating multi-omics data and machine learning, we developed a 14-gene signature that reflects key biological processes including extracellular matrix remodeling and immune microenvironment modulation. The BMS demonstrated consistent predictive accuracy across multiple cohorts, with high-risk patients showing significantly poorer prognosis compared to low-risk patients. Functional validation identified FREM1 as a potential tumor suppressor, providing mechanistic insights into basement membrane biology. While retrospective in nature, our findings highlight the clinical potential of BMS for risk assessment and treatment guidance. Future studies should focus on prospective validation, mechanistic exploration of basement membrane-immune interactions, and therapeutic targeting of identified pathways to improve patient outcomes. This work advances our understanding of breast cancer heterogeneity and provides a foundation for more personalized management strategies.

## Supplementary Information

Supplementary file 1. Table 1. Relationship between predicted model-derived risk scores and clinical characteristics in breast cancer.

Supplementary file 2. Table 2. Association between FREM1 expression levels and clinical characteristics in breast cancer.

Supplementary file 3. Figure 1. Prognostic nomograms and calibration curves for survival outcomes in breast cancer. Overall Survival (OS) Nomogram and Calibration Curve. Progression-Free Survival (PFS) Nomogram and Calibration Curve. Disease-Free Survival (DFS) Nomogram and Calibration Curve. Disease-Specific Survival (DSS) Nomogram and Calibration Curve

Supplementary file 4. Figure 2. Prognostic and differential expression analysis of six candidate genes (*SEMA3B, ITGAX, FREM1, ADAM9, ADAMTS8*, and *UNC5A*) in breast cancer. (A) Kaplan-Meier survival curves for overall survival (OS) stratified by high (red) and low (blue) expression levels of each gene in the TCGA-BRCA cohort. (B) Comparative analysis of gene expression between primary breast tumor tissues (red) and adjacent normal tissues (blue) from TCGA-BRCA.

Supplementary file 5. Figure 3. CIBERSORT analysis revealing the relationship between FREM1 expression levels and the infiltration of 22 immune cell populations in breast cancer.

## Data Availability

The TCGA-Breast Invasive Carcinoma (BRCA) dataset was obtained from the Genomic Data Commons (GDC) Data Portal (https://portal.gdc.cancer.gov/), and the following Gene Expression Omnibus (GEO) datasets were analyzed: GSE131769, GSE148673, and GSE176078 (https://www.ncbi.nlm.nih.gov/geo/). All datasets used in this study are publicly available and can be accessed directly via the provided links. Additional data (if not publicly available) are available from the corresponding author upon reasonable request.
