# Supplementary material for: FREM1 serves as a novel therapeutic target in breast cancer through basement membrane-based prognostic modeling with integrated bioinformatics and experimental validation
Source: Discov Oncol. 2025 Dec 1;17:15. doi: 10.1007/s12672-025-04117-3 (PMC12770021; doi:10.1007/s12672-025-04117-3)
Supplement: Supplementary file 1 — Table 1. Relationship between predicted model-derived risk scores and clinical characteristics in breast cancer. [file 12672_2025_4117_MOESM1_ESM.docx]

**Supplementary Table 1. Relationship between predicted model-derived risk scores and clinical characteristics in breast cancer.**

| Characteristic | Risk Score Level | | p-value |
| --- | --- | --- | --- |
|  | High | Low |  |
| Age |  |  |  |
| Mean(SD) | 56.4(11.7) | 60.5(14.2) | 0.001 |
| T |  |  | 0.001 |
| T1 | 161 | 115 |  |
| T2 | 299 | 319 |  |
| T3 | 68 | 70 |  |
| T4 | 8 | 27 |  |
| Tx | 1 | 2 |  |
| N |  |  | 0.035 |
| N0 | 276 | 233 |  |
| N1 | 173 | 178 |  |
| N2 | 48 | 68 |  |
| N3 | 34 | 41 |  |
| Nx | 6 | 13 |  |
| M |  |  | 0.005 |
| M0 | 435 | 458 |  |
| M1 | 6 | 13 |  |
| Mx | 96 | 62 |  |
| Stage |  |  | 0.001 |
| I | 111 | 71 |  |
| II | 310 | 301 |  |
| III | 108 | 137 |  |
| IV | 6 | 13 |  |
| X | 2 | 11 |  |
| Status |  |  | 0.007 |
| Alive | 487 | 449 |  |
| Dead | 61 | 92 |  |
