# Supplementary material for: FREM1 serves as a novel therapeutic target in breast cancer through basement membrane-based prognostic modeling with integrated bioinformatics and experimental validation
Source: Discov Oncol. 2025 Dec 1;17:15. doi: 10.1007/s12672-025-04117-3 (PMC12770021; doi:10.1007/s12672-025-04117-3)
Supplement: Supplementary file 2 — Table 2. Association between FREM1 expression levels and clinical characteristics in breast cancer. [file 12672_2025_4117_MOESM2_ESM.docx]

**Supplementary Table 2. Association between FREM1 expression levels and clinical characteristics in breast cancer.**

| Characteristic | FREM1 Expression Level | | p-value |
| --- | --- | --- | --- |
|  | High | Low |  |
| Age |  |  |  |
| Mean(SD) | 56(12.2) | 60.3(13.9) | 0.002 |
| T |  |  | 0.001 |
| T1 | 163 | 117 |  |
| T2 | 287 | 342 |  |
| T3 | 83 | 55 |  |
| T4 | 14 | 25 |  |
| Tx | 1 | 2 |  |
| N |  |  | 0.495 |
| N0 | 257 | 255 |  |
| N1 | 179 | 181 |  |
| N2 | 64 | 55 |  |
| N3 | 42 | 36 |  |
| Nx | 6 | 14 |  |
| M |  |  | 0.012 |
| M0 | 446 | 454 |  |
| M1 | 5 | 16 |  |
| Mx | 92 | 70 |  |
| Stage |  |  | 0.017 |
| I | 109 | 73 |  |
| II | 286 | 330 |  |
| III | 139 | 109 |  |
| IV | 5 | 14 |  |
| X | 6 | 7 |  |
| Status |  |  | 0.007 |
| Alive | 487 | 449 |  |
| Dead | 61 | 92 |  |
